# Decreased free d-aspartate levels are linked to enhanced d-aspartate oxidase activity in the dorsolateral prefrontal cortex of schizophrenia patients

**DOI:** 10.1038/s41537-017-0015-7

**Published:** 2017-04-06

**Authors:** Tommaso Nuzzo, Silvia Sacchi, Francesco Errico, Simona Keller, Orazio Palumbo, Ermanno Florio, Daniela Punzo, Francesco Napolitano, Massimiliano Copetti, Massimo Carella, Lorenzo Chiariotti, Alessandro Bertolino, Loredano Pollegioni, Alessandro Usiello

**Affiliations:** 1 Laboratory of Behavioral Neuroscience, Ceinge Biotecnologie Avanzate, 80145 Naples, Italy; 2 Department of Environmental, Biological and Pharmaceutical Sciences and Technologies, Second University of Naples (SUN), 81100 Caserta, Italy; 3 Department of Biotechnology and Life Sciences, University of Insubria, 21100 Varese, Italy; 4 The Protein Factory Research Center, Politecnico di Milano and University of Insubria, 20133 Milan, Italy; 5 Department of Molecular Medicine and Medical Biotechnology, University of Naples “Federico II”, 80131 Naples, Italy; 6 Endocrinology and Molecular Oncology Institute (I.E.O.S.), National Research Council (C.N.R.), 80131 Naples, Italy; 7 Medical Genetics Unit, IRCCS Casa Sollievo della Sofferenza, 71013 San Giovanni Rotondo, Foggia Italy; 8 Unit of Biostatistics, IRCCS Casa Sollievo della Sofferenza, 71013 San Giovanni Rotondo, Foggia Italy; 9 Department of Basic Medical Science, Neuroscience, and Sense Organs, University of Bari “Aldo Moro”, 70124 Bari, Italy

## Abstract

It is long acknowledged that the *N*-methyl d-aspartate receptor co-agonist, d-serine, plays a crucial role in several *N*-methyl d-aspartate receptor-mediated physiological and pathological processes, including schizophrenia. Besides d-serine, another free d-amino acid, d-aspartate, is involved in the activation of *N*-methyl d-aspartate receptors acting as an agonist of this receptor subclass, and is abundantly detected in the developing human brain. Based on the hypothesis of *N*-methyl d-aspartate receptor hypofunction in the pathophysiology of schizophrenia and considering the ability of d-aspartate and d-serine to stimulate *N*-methyl d-aspartate receptor-dependent transmission, in the present work we assessed the concentration of these two d-amino acids in the *post-mortem* dorsolateral prefrontal cortex and hippocampus of patients with schizophrenia and healthy subjects. Moreover, in this cohort of *post-mortem* brain samples we investigated the spatiotemporal variations of d-aspartate and d-serine. Consistent with previous work, we found that d-aspartate content was selectively decreased by around 30% in the dorsolateral prefrontal cortex, but not in the hippocampus, of schizophrenia-affected patients, compared to healthy subjects. Interestingly, such selective reduction was associated to greater (around 25%) cortical activity of the enzyme responsible for d-aspartate catabolism, d-aspartate oxidase. Conversely, no significant changes were found in the methylation state and transcription of *DDO* gene in patients with schizophrenia, compared to control individuals, as well as in the expression levels of serine racemase, the major enzyme responsible for d-serine biosynthesis, which also catalyzes aspartate racemization. These results reveal the potential involvement of altered d-aspartate metabolism in the dorsolateral prefrontal cortex as a factor contributing to dysfunctional *N*-methyl d-aspartate receptor-mediated transmission in schizophrenia.

## NMDA receptor: Enzyme breaks down ion channel activator in schizophrenic brain

Altered metabolism of an amino acid activator of ion channels in the brain could explain dysfunctional nerve signaling in schizophrenia. Researchers in Italy led by Alessandro Usiello from Ceinge Biotecnologie Avanzate and Loredano Pollegioni from the University of Insubria measured the levels of two amino acids—D-aspartate and D-serine—in post-mortem tissues taken from two brain regions of patients with and without schizophrenia. Both amino acids activate the N-methyl D-aspartate receptor, which is known to be less active in people with schizophrenia. The researchers found a mild increase in D-serine levels but a major decrease in D-aspartate in the schizophrenia patients’ dorsolateral prefrontal cortex (DLPFC), a memory and reasoning part of the brain, but not in the hippocampus. They also documented a greater activity of the enzyme responsible for D-aspartate breakdown in the DLPFC.

## Introduction

d-serine (d-Ser) and d-aspartate (d-Asp) are the only two free d-amino acids occurring at substantial levels in the mammalian brain. d-Ser is present at high concentrations throughout prenatal and postnatal life, especially in the forebrain regions^1^ where *N*-methyl d-aspartate receptors (NMDARs) are abundant. Together with glycine, d-Ser activates the NMDAR co-agonist site and plays a crucial role in NMDAR-mediated physiological and pathological processes, including those involved in schizophrenia (SCZ).^2, 3^ In this regard, abnormal d-Ser content and metabolism have been observed in the cerebrospinal fluid and serum of subjects with SCZ.^4–6^ As in humans, several findings collected in preclinical models indicated an involvement of this d-amino acid in SCZ-related phenotypes. For instance, reduced d-Ser levels due to the ablation of the gene *Serine racemase* (*Srr*)^7^ encoding the enzyme responsible for d-Ser biosynthesis, produce in mutant mice NMDAR-related morphological and behavioral deficits reminiscent of those occurring in SCZ.^3^ On the other hand, d-Ser supplementation in animal models was showed to be effective in improving a range of SCZ-related cognitive, sensorimotor, and social deficits.^2, 3^

In contrast to d-Ser, d-Asp undergoes a strict developmental regulation since it occurs at high levels during embryonic and early postnatal phases, and markedly drops thereafter.^1, 8^ Remarkably, in the human prefrontal cortex (PFC) at 14 weeks of gestation d-Asp levels even exceed those of the corresponding l-form before the drastic postnatal decrease.^1^ The peculiar temporal pattern of d-Asp occurrence depends on the postnatal onset of d-aspartate oxidase (DDO or DASPO, EC 1.4.3.1) activity, a peroxisomal flavoenzyme that selectively catabolises d-Asp.^9^ In the mouse brain, the age-dependent increase in DDO enzymatic activity is paralleled by concomitant rise in *Ddo* mRNA levels that, in turn, is associated to progressive *Ddo* gene demethylation in the putative regulatory region surrounding the transcription start site.^8^

Several studies have so far indicated that d-Asp activates NMDARs through its binding at the glutamate (Glu) site of this receptor.^9, 10^ Consistent with the knowledge that hypofunction of NMDARs elicits SCZ-like symptoms in humans and preclinical models,^11, 12^ it has been shown that higher cerebral levels of d-Asp in mice are protective against prepulse inhibition (PPI) deficits and abnormal circuits activation induced by the psychotomimetic drug phencyclidine (PCP). In addition, non-physiological d-Asp levels improve cognition,^13–15^ enhance cortico-hippocampal connectivity,^16^ and increase structural and functional synaptic plasticity in cortical and hippocampal pyramidal neurons.^17^ Translation of these preclinical studies to healthy humans indicated that the CC genotype of the *DDO* polymorphism rs3757351, predicting reduced *DDO* mRNA expression in the *post-mortem* PFC, is associated with prefrontal phenotypes relevant to SCZ, such as greater gray matter volume and enhanced activity during working memory tasks.^17^ Moreover, evidence obtained in human *post-mortem* samples have shown substantially decreased content of d-Asp in patients with SCZ, compared to non-psychiatric controls.^18^

Based on this previous finding, we used another collection of *post-mortem* dorsolateral prefrontal cortex (DLPFC) and hippocampus samples of SCZ-affected patients and control individuals to investigate whether SCZ diagnosis affects the levels of d-Asp and its regulation by DDO by assessing *DDO* gene copy number, *DDO* gene methylation, *DDO* mRNA expression and DDO enzymatic activity. These brain samples were also analyzed for the content of d-Ser and for the expression levels of SRR protein, which is not only responsible for d-Ser production but has been also recently proposed to contribute to d-Asp biosynthesis.^19, 20^

## Results

### Age-related distribution of d-aspartate and d-serine in the brain of patients with SCZ and healthy subjects

**Fig. 1:**
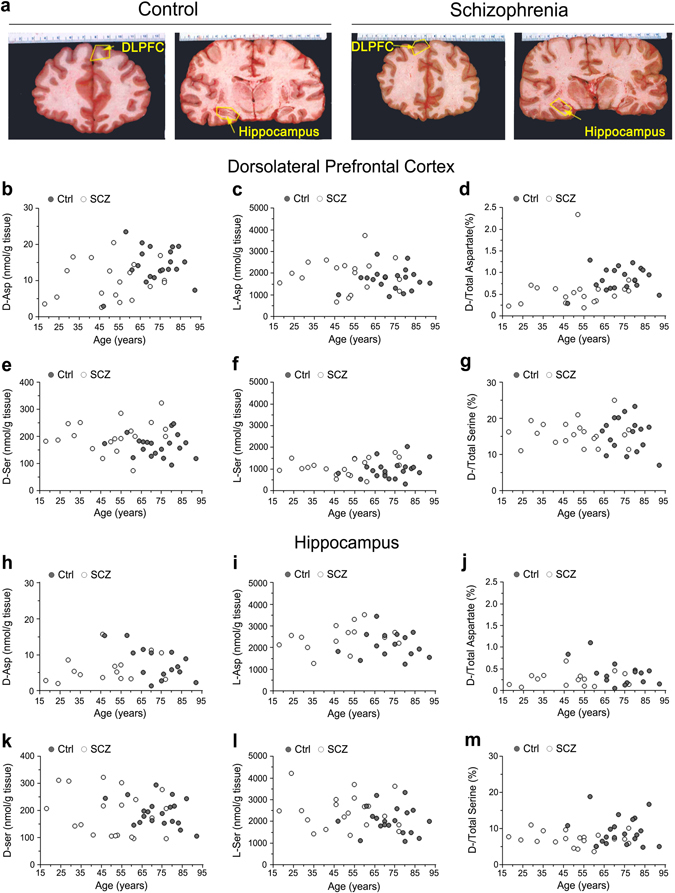
Age-related changes in d-aspartate and d-serine levels between patients with schizophrenia and control subjects in the *post-mortem* dorsolateral prefrontal cortex and hippocampus. **a** Representative images of the dorsolateral prefrontal cortex (*DLPFC*) and hippocampus dissected from *post-mortem* brains of healthy (control, Ctrl) and schizophrenia-affected individuals (*SCZ*). The *dot* plots represent the variations across lifespan in the content of free **b**, **h** d-aspartate and **e**, **k** d-serine, **c**, **i** l-aspartate and **f**, **l** l-serine, and **d**, **g**, **j**, **m** d-/total amino acids in the **b**–**g** dorsolateral prefrontal cortex (*DLPFC*) and **h–m** hippocampus of non-psychiatric individuals (DLPCF, *n* = 20; hippocampus, *n* = 15) and patients with schizophrenia (DLPCF, *n* = 19; hippocampus, *n* = 15). In each sample, all the amino acids were detected in a single run by HPLC and expressed as nmol/g of tissue, while the ratios are expressed as percentage (%)

It has been previously reported that the content of d-Asp in the *post-mortem* PFC of healthy subjects drastically declines from prenatal to postnatal stage, and remains low during adulthood.^1^ However, there is no evidence relative to the temporal variations of d-Asp content in the brain of SCZ-affected patients. Therefore, we analyzed the changes in d-Asp levels across adult life in the *post-mortem* DLPFC and hippocampus dissected from individuals with SCZ, compared to controls (representative images of these brain regions are shown in Fig. 1a). In parallel to d-Asp, we also assessed the age-span occurrence of d-Ser.

We found that d-Asp levels do not significantly change along adulthood in both the DLPFC and hippocampus of control subjects (DLPFC: *r* = 0.0368, *p* = 0.8733; hippocampus: *r* = −0.4924, *p* = 0.0620; Spearman correlation) and SCZ-affected patients (DLPFC: *r* = 0.1397, *p* = 0.5685; hippocampus: *r* = 0.2308, *p* = 0.4050) (Fig. 1b, h), although there is a significant difference in the mean age of the two diagnosis groups (*p* < 0.01, Supplementary Table 1). Similarly, in both brain regions the l-Asp levels (Ctrl: DLPFC, *r* = 0.0557, *p* = 0.8156; hippocampus, *r* = −0.0841, *p* = 0.7551; SCZ: DLPFC, *r* = 0.0053, *p* = 0.9829; hippocampus, *r* = 0.3488, *p* = 0.2016) and the d-Asp/total Asp ratio (Ctrl: DLPFC, *r* = 0.1153, *p* = 0.6285; hippocampus, *r* = −0.3133, *p* = 0.2481; SCZ: DLPFC, *r* = 0.1751, *p* = 0.4734; hippocampus, *r* = 0.1395, *p* = 0.6177) were unaffected by the age of the individuals, independently from their clinical diagnosis (Fig. 1c, d, i, and j).

d- and l-Ser were concomitantly analyzed on the same samples. The levels of both enantiomers remained constant during adulthood, in both the DLPFC and hippocampus of healthy individuals (d-Ser: DLPFC, *r* = −0.0685, *p* = 0.7742; hippocampus, *r* = −0.2266, *p* = 0.3368; l-Ser: DLPFC, *r* = 0.2665, *p* = 0.2561; hippocampus, *r* = −0.1280, *p* = 0.5908) and SCZ patients (d-Ser: DLPFC, *r* = 0.2966, *p* = 0.2042; hippocampus, *r* = −0.2597, *p* = 0.2689; l-Ser: DLPFC, *r* = 0.2958, *p* = 0.2054; hippocampus, *r* = −0.0858, *p* = 0.7191) (Fig. 1e, f, k, and l). Accordingly, the d-Ser/total Ser ratio did not change in the two brain regions during adult age in both clinical conditions (Ctrl: DLPFC, *r* = −0.1905, *p* = 0.4211; hippocampus, *r* = −0.1114, *p* = 0.6401; SCZ: DLPFC, *r* = −0.1046, *p* = 0.6607; hippocampus, *r* = −0.1626, *p* = 0.4934) (Fig. 1g, m).

Overall, these data excluded significant age-dependent variations of d-Asp and d-Ser levels in the DLPFC and hippocampus associated to the diagnosis of SCZ.

### Selective reduction of d-aspartate levels in the DLPFC of patients with SCZ

**Fig. 2:**
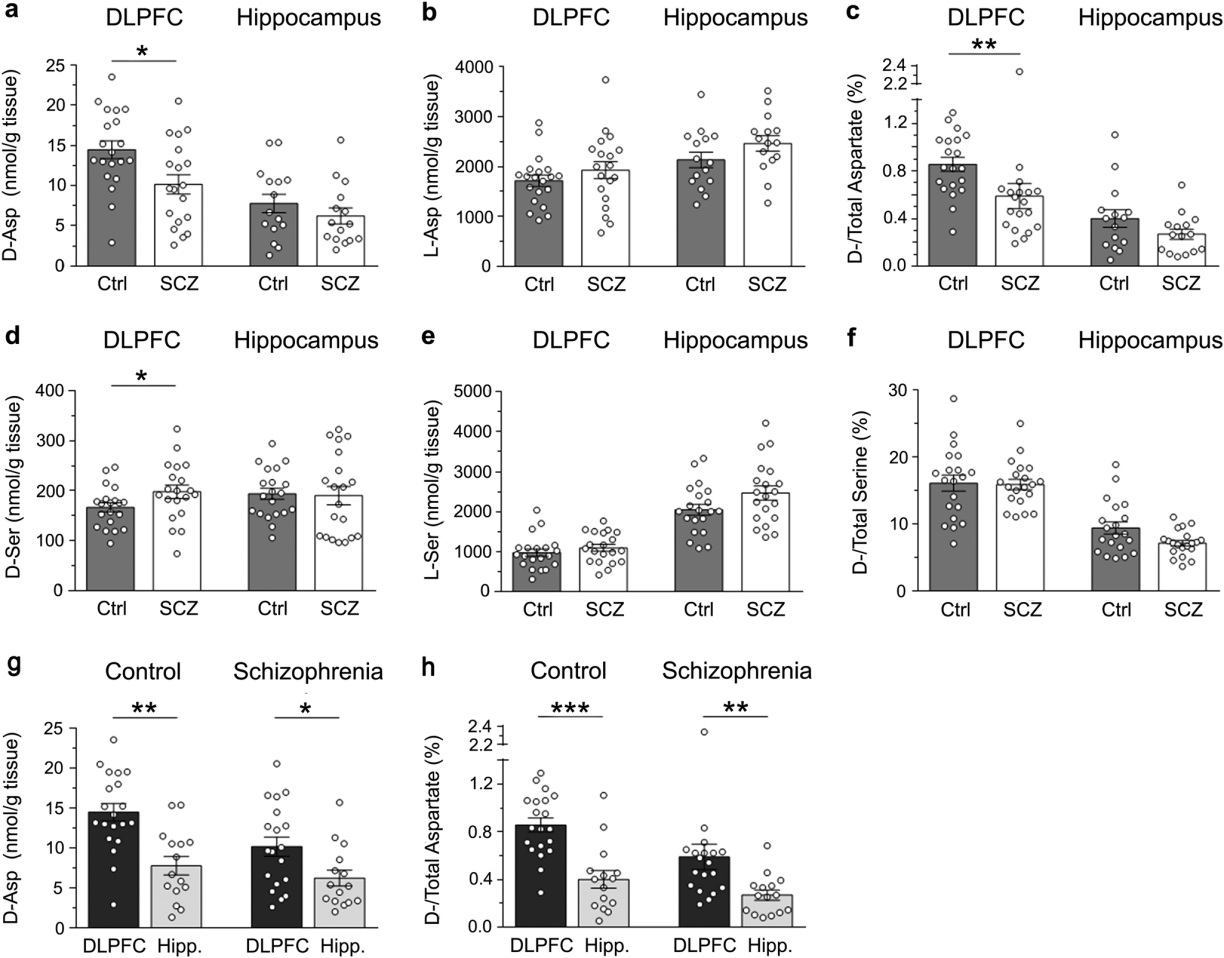
Changes in d-aspartate and d-serine levels between patients with schizophrenia and control subjects in the *post-mortem* dorsolateral prefrontal cortex and hippocampus. **a** d-aspartate, **b** l-aspartate, **d** d-serine and **e** l-serine levels, **c** d-aspartate/total aspartate and **f** d-serine/total serine are compared between non-psychiatric individuals (control, Ctrl) and patients with schizophrenia (*SCZ*) in the *post-mortem* dorsolateral prefrontal cortex (*DLPFC*) (Ctrl, *n* = 20; SCZ, *n* = 19) and hippocampus (Ctrl, *n* = 15; SCZ, *n* = 15). **g, h** d-aspartate levels and d/total aspartate ratio were also compared between the DLPFC and hippocampus (Hipp.) of non-psychiatric individuals (DLPCF, *n* = 20; hippocampus, *n* = 15) and patients with schizophrenia (DLPCF, *n* = 19; hippocampus, *n* = 15). In each sample, all the amino acids were detected in a single run by HPLC and expressed as nmol/g of tissue, while the ratios are expressed as percentage (%). *p < 0.05; **p < 0.01; ***p < 0.0001 (Mann–Whitney test). *Dots* represent the single subjects’ values while *bars* illustrate the means ± SEM

We evaluated whether the reduction (∼40%) in d-Asp levels previously found in the *post-mortem* PFC of patients with SCZ (Institute of Psychiatry, King’s College London, UK)^18^ might be replicated in a new cohort of brain tissues from SCZ and control cases (Human Brain and Spinal Fluid Research Center, Los Angeles, CA, USA). In line with previous data, a significant (∼30%) reduction in d-Asp levels was detected in the DLPFC of SCZ-affected patients, compared to healthy individuals (*p* < 0.05, Mann–Whitney test) (Fig. 2a and Supplementary Table 2). On the other hand, no difference in l-Asp levels was apparent between diagnoses (*p* > 0.05) (Fig. 2b and Supplementary Table 2). Accordingly, the d-Asp/total Asp ratio was significantly lower in patients with SCZ, compared to controls (*p* < 0.01) (Fig. 2c and Supplementary Table 2). Differently from DLPFC, in the hippocampus we found no statistically significant change in d-Asp and l-Asp levels between SCZ-affected patients and control subjects (*p* > 0.05 for both) (Fig. 2a, b and Supplementary Table 2), as well as in the d-Asp/total Asp ratio (*p* > 0.05) (Fig. 2c and Supplementary Table 2).

In addition, we found a mild increase in d-Ser content (∼15%) in the DLPFC of SCZ patients, compared to control subjects (*p* < 0.05), while no difference was found in the hippocampus of SCZ-affected patients and healthy subjects (*p* > 0.05) (Fig. 2d and Supplementary Table 2). However, in both brain regions and diagnostic groups analyzed, we detected comparable l-Ser levels and d-Ser/total Ser ratio (*p* > 0.05) (Fig. 2e, f and Supplementary Table 2).

Finally, we assessed whether the diagnosis of SCZ could influence the regional differences of d-Asp levels occurring between the DLPFC and the hippocampus. We found that the d-Asp content was significantly higher in the DLPFC than in the hippocampus, in both healthy and SCZ-affected individuals (Ctrl: *p* < 0.01, SCZ: *p* < 0.05; Mann–Whitney test) (Fig. 2g and Supplementary Table 3). Similarly, the d-Asp/total Asp ratio was significantly higher in the DLPFC than in the hippocampus, regardless of the diagnosis (Ctrl: *p* < 0.0001; SCZ: *p* < 0.05) (Fig. 2h and Supplementary Table 3).

### *DDO* gene methylation and *DDO* mRNA expression in the DLPFC and hippocampus of patients with SCZ and healthy subjects

**Fig. 3:**
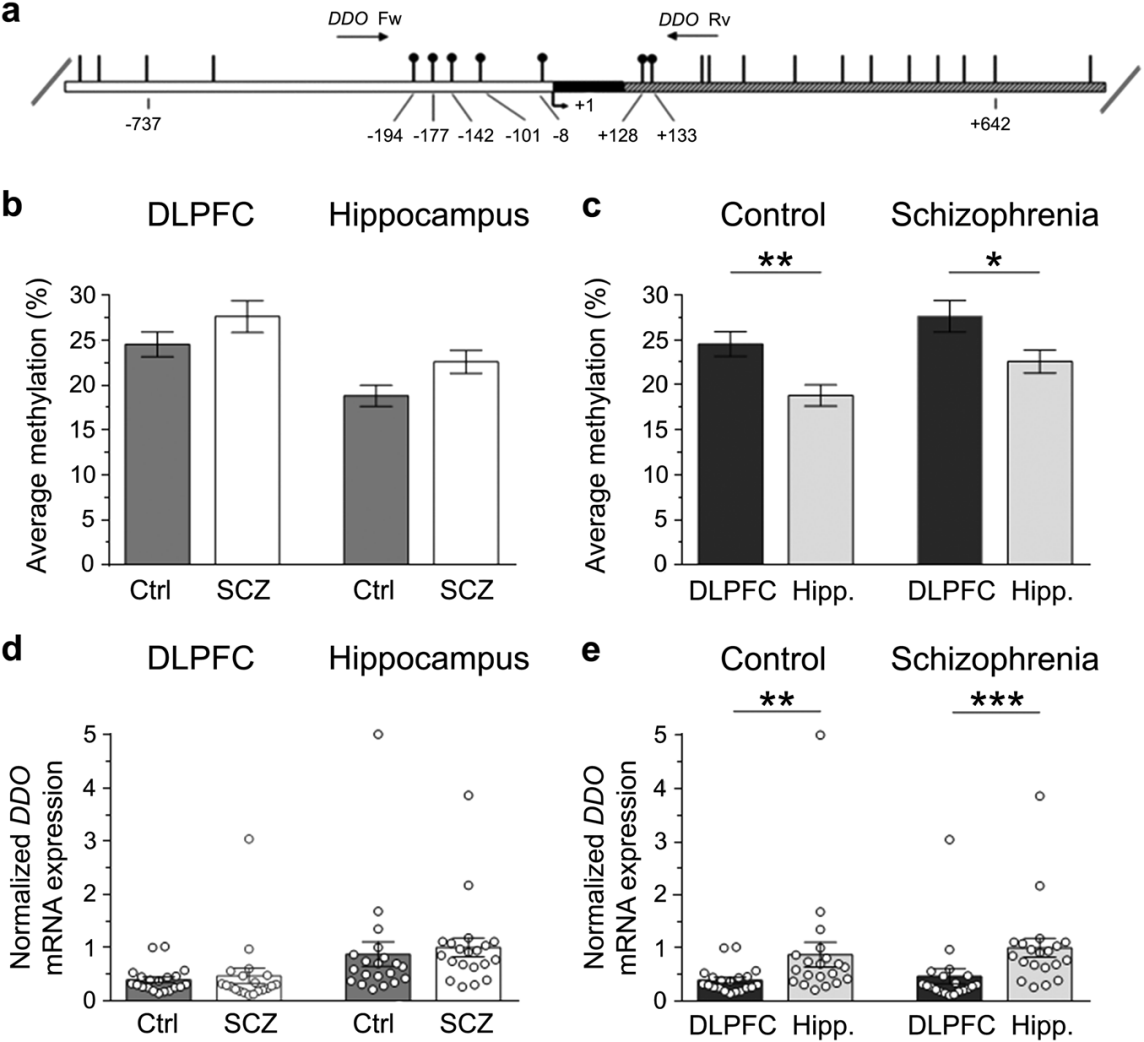
*DDO* gene methylation and *DDO* mRNA expression in the dorsolateral prefrontal cortex (*DLPFC*) and hippocampus (Hipp.) of schizophrenia-affected patients (*SCZ*) and control (Ctrl) subjects. **a** Structure of the putative promoter of the human *DDO* gene. *Arrow* indicates the transcription start site (TSS,+1). *White*, *black*, and *gray* boxes represent, respectively, the putative regulatory upstream region, the exon 1 and the first inton. Position of primers used for bisulfite analysis is indicated by *arrows* at the top of the map (*Ddo*Fw, *Ddo*Rv). *Vertical bars* represent the relative positions of each CpG site. *Black circles* represent the CpG sites analyzed. **b**, **c** Average methylation of the seven CpG sites analyzed is compared **b** between the two diagnosis groups (DLPFC: Ctrl, *n* = 20; SCZ, *n* = 20−hippocampus: Ctrl, *n* = 20; SCZ, *n* = 19), or **c** between the two brain regions (Ctrl: DLPFC, *n* = 20; hippocampus, *n* = 20−SCZ: DLPFC, *n* = 20; hippocampus, *n* = 19). The percent (%) of methylation represents the average of the seven CpG sites contained in the analyzed region. **d, e** *DDO* gene transcription is compared **d** between the two diagnosis groups (DLPFC: Ctrl, *n* = 20; SCZ, *n* = 20−hippocampus: Ctrl, *n* = 20; SCZ, *n* = 20), and **e** between the two brain regions (Ctrl: DLPFC, *n* = 20; hippocampus, *n* = 20−SCZ: DLPFC, *n* = 20; hippocampus, *n* = 20). The *DDO* mRNA expression is normalized to the mean of two housekeeping genes and expressed as arbitrary units. *p < 0.05, **p < 0.01,***p < 0.0001 (Mann–Whitney test). *Dots* represent the single subjects’ values while *bars* illustrate the means ± SEM

We recently reported that the state of methylation of the regulatory region of the *DDO* gene spanning across the transcriptional start site controls the postnatal rise of *DDO* mRNA levels in the mouse brain thus predicting, in turn, the content of d-Asp.^8^ Based on this finding, we evaluated whether epigenetic modification at the *DDO* locus was affected by the diagnosis of SCZ. To this aim, a bisulfite-seq analysis in the *post-mortem* DLPFC and hippocampus of SCZ-affected patients and healthy subjects was performed. We analyzed the average methylation state of the seven CpG sites (−194, −177, −142, −101, −8, +128 and +133) lying in the region of the human *DDO* surrounding the transcription start site spanning from −199 to +177 nucleotide positions (Fig. 3a). No significant alteration in *DDO* gene methylation state was observed in the DLPFC of the two diagnostic groups (mean values: Ctrl, 24.5 ± 1.4%; SCZ, 27.6 ± 1.8%; *p* > 0.05, Mann–Whitney test) while a significant, moderate increase was detected in the hippocampus of SCZ-affected patients, compared to healthy individuals (control, 18.8 ± 1.2%; SCZ, 22.6 ± 1.3%; *p* < 0.05) (Fig. 3b).

Next, we analyzed the regional variations in the *DDO* gene methylation profile in both healthy and SCZ-affected subjects. The average methylation state of this gene was significantly higher in the DLPFC than in the hippocampus in both control individuals (mean values: DLPFC, 24.5 ± 1.4%; hippocampus, 18.8 ± 1.2%; *p* < 0.01) and in SCZ-affected patients (mean values: DLPFC, 27.6 ± 1.8%; hippocampus, 22.6 ± 1.3%; *p* < 0.05) (Fig. 3c).

We then examined the *DDO* gene transcription in the *post-mortem* brain of patients with SCZ and relative controls. Interestingly, quantitative reverse transcription PCR (qRT-PCR) analysis did not reveal any significant difference in *DDO* mRNA levels between SCZ-affected and healthy individuals, in both the brain structures analyzed (mean values, DLPFC: Ctrl, 0.39 ± 0.05; SCZ, 0.47 ± 0.14; *p* > 0.05; hippocampus: Ctrl, 0.87 ± 0.23; SCZ, 1.00 ± 0.18; *p* > 0.05) (Fig. 3d).

On the other hand, consistently with an influence of *DDO* gene methylation on mRNA expression,^8^ we found reduced levels of *DDO* transcript in the DLPFC, compared to the hippocampus, independently of the clinical diagnosis (mean values, Ctrl: DLPFC, 0.39 ± 0.05; hippocampus, 0.87 ± 0.23; *p* < 0.01; SCZ: DLPFC, 0.47 ± 0.14; hippocampus, 1.00 ± 0.18; *p* < 0.0001) (Fig. 3e). Interestingly, regional changes in the state of methylation and in the transcription of *DDO* gene found in the human brain seem to be conserved across species since we found similar epigenetic and gene transcription variations in the mouse ortholog *Ddo* gene (see Supplementary Fig. 1).

### DDO enzymatic activity in the DLPFC and hippocampus of patients with SCZ and healthy subjects

**Fig. 4:**
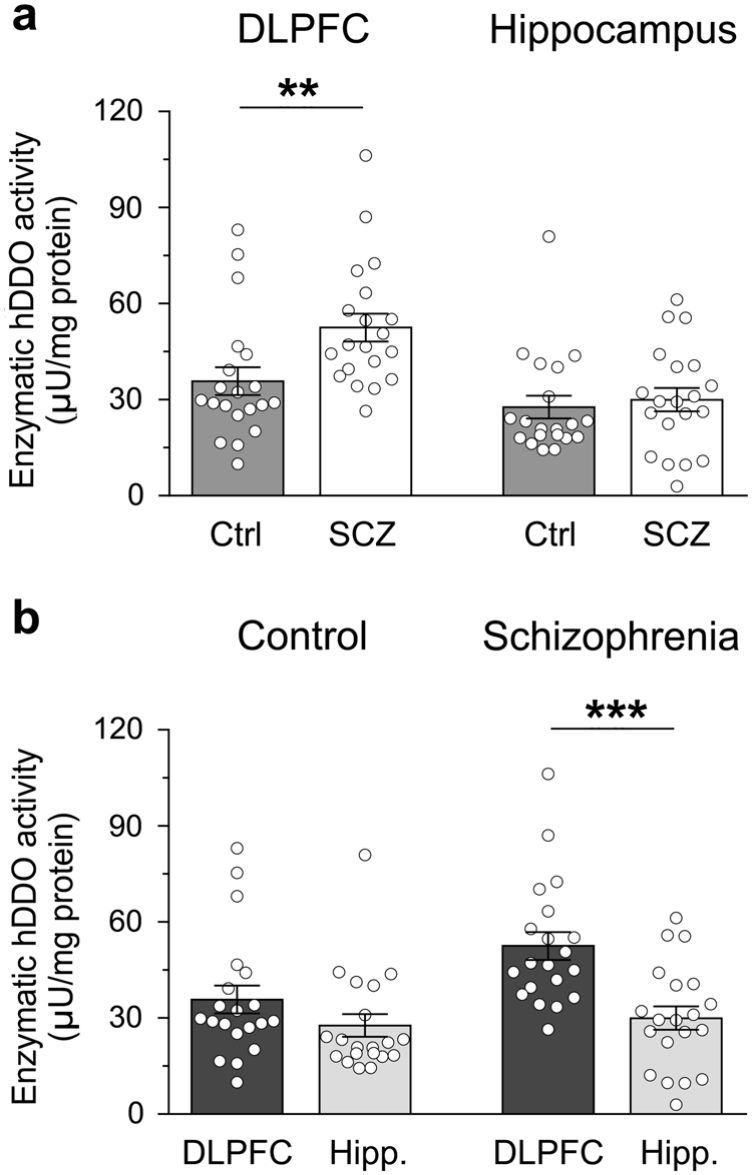
Enzymatic activity of human d-aspartate oxidase (*hDDO*) in the dorsolateral prefrontal cortex (*DLPFC*) and hippocampus (*Hipp*.) of patients with schizophrenia (*SCZ*) and control (Ctrl) subjects. DDO enzymatic activity is analyzed **a** between the two diagnosis groups (DLPFC: Ctrl, *n* = 20; SCZ, *n* = 20−hippocampus: Ctrl, *n* = 20; SCZ, *n* = 19), and **b** between the two brain regions (Ctrl: DLPFC, *n* = 20; hippocampus, *n* = 20−SCZ: DLPFC, *n* = 20; hippocampus, *n* = 20). The enzymatic activity of DDO is expressed as μU/mg of total proteins. **p < 0.01, ***p < 0.0001 (Mann–Whitney test). *Dots* represent the single subjects’ values while *bars* illustrate the means ± SEM

In order to link altered d-Asp levels to abnormal DDO regulation, the activity of the enzyme was evaluated in the *post-mortem* brain of SCZ-affected patients and healthy individuals. Interestingly, in line with reduced content of d-Asp found in the DLPFC of SCZ-affected subjects (Fig. 2a), in this brain region of patients we found increased DDO activity, compared to controls (mean values: Ctrl, 35.8 ± 4.3 μU/mg protein; SCZ, 52.5 ± 4.4 μU/mg protein; *p* < 0.01, Mann–Whitney test) (Fig. 4a). On the other side, no significant changes in the levels of DDO activity were observed in the hippocampus of patients, compared to healthy subjects (mean values: Ctrl, 27.7 ± 3.6 μU/mg protein; SCZ, 30.0 ± 3.7 μU/mg protein; *p* > 0.05) (Fig. 4a).

In spite of the different transcriptional regulation of *DDO* found between the DLPFC and hippocampus, the DDO enzymatic activity was not significantly changed between these two brain regions in control subjects (mean values: DLPFC, 35.8 ± 4.3 μU/mg protein, hippocampus, 27.7 ± 3.6 μU/mg protein; *p* > 0.05) while in SCZ-affected individuals it was significantly higher in the DLPFC than in the hippocampus (mean values: DLPFC, 52.5 ± 4.4 μU/mg protein; hippocampus, 30.0 ± 3.7 μU/mg protein; *p* < 0.0001) (Fig. 4b).

### The enzymatic activity of human DDO (hDDO) is not affected by widely used first- and second-generation antipsychotics

Since the use of antipsychotic medications may represent a potential confounding factor for the analysis of d-Asp levels in the *post-mortem* SCZ brains, we tested in vitro whether some of the widely used antipsychotics may interfere with recombinant hDDO activity. To this aim, we tested two first-generation antipsychotics, haloperidol and chlorpromazine, and a second-generation antipsychotic, clozapine. Overall, the activity assays indicated that these compounds have no effect on hDDO activity, even when their concentration is in the low millimolar range (Table 1). These results suggest that the exposure to common antipsychotic drugs is unlikely to affect the catabolism of d-Asp in the brain of patients with SCZ.

**Table 1:** Inhibition assays showing the relative activity (%) of recombinant human DDO (hDDO) in the presence of different concentrations of antipsychotic drugs

		Concentration (µM)			
Antipsychotic	Chemical structure	1	10	100	1000
Chlorpromazine		100	100	100	>90
Haloperidol		>95	>95	100	100
Clozapine		100	100	>95	>95

### Lack of difference in serine racemase protein expression in the DLPFC and hippocampus between patients with SCZ and healthy subjects

**Fig. 5:**
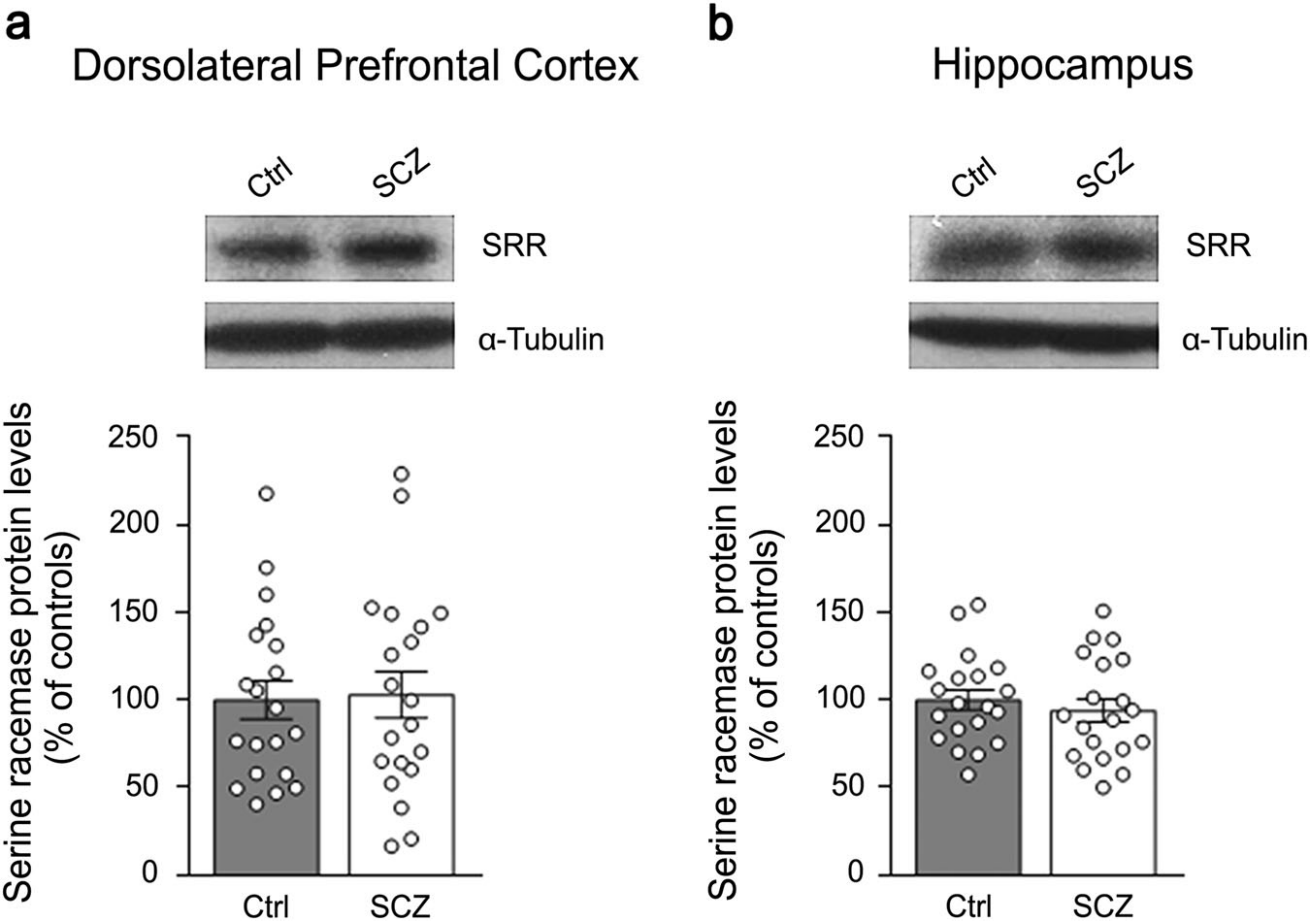
Serine racemase protein levels in the *post-mortem* dorsolateral prefrontal cortex and hippocampus of patients with schizophrenia. The expression of serine racemase (SRR) was assessed by western blotting in the **a** dorsolateral prefrontal cortex and **b** hippocampus of schizophrenia-affected patients (*SCZ*) and control (Ctrl) individuals (*n* = 20 per brain region and diagnosis). The variations of SRR protein levels in patients are expressed as percentage (%) of the control group (healthy subjects). α-Tubulin protein was used to normalize for variations in loading and transfer. Representative blots comparing the two diagnostic groups are shown above the graphs. *Dots* represent the single subjects’ values while *bars* illustrate the means ± SEM

Recent findings indicated that the mammalian serine racemase (SRR), the primary enzyme responsible for brain d-Ser production,^7^ could be also involved in d-Asp biosynthesis.^19, 20^ Indeed, *Srr* knockout mice display not only a strong d-Ser levels decrease (~90%) in their brain,^7^ but also a substantial reduction (50–65%) in d-Asp amount in the frontal cortex and hippocampus,^19, 20^ where SRR is highly expressed.^7^ Based on this knowledge, and consistent with the neurochemical alteration of d-Asp levels found in SCZ brain samples, we measured SRR protein levels in patients with SCZ and control subjects by western blotting approach. Our data revealed that the protein amount of SRR is comparable between SCZ-affected patients and healthy individuals, both in the DLPFC and hippocampus (*p* > 0.05, Mann–Whitney test) (Figs. 5a, b). Such result highlights that the reported decreased d-Asp concentration found in the DLPFC of SCZ patients is not associated to alterations in SRR protein expression.

## Discussion

A large bulk of findings supports the influence of NMDAR hypofunction in the pathogenesis of SCZ.^11, 12^ Accordingly, complex dysfunctions in glutamatergic transmission have been reported at both presynaptic and postsynaptic level,^11^ including altered mRNA and protein expression of NMDAR subunits in the *post-mortem* SCZ brain.^21, 22^ Besides direct alterations in NMDARs, evidence achieved over the last 15 years suggested that altered metabolism of the co-agonist d-Ser may contribute to the pathophysiological mechanisms leading to SCZ.^2, 3^ Accordingly, reduced d-Ser levels have been detected in the CSF^4, 5^ and serum,^6, 23, 24^ but not in the *post-mortem* brain tissues of patients suffering from SCZ.^4, 25^ Furthermore, different clinical studies have demonstrated that d-Ser supplementation could be beneficial for SCZ symptoms.^3, 26^ In line with previous findings,^4, 25^ herein we detected comparable or slightly higher d-Ser levels, respectively in the *post-mortem* hippocampus and DLPFC of patients with SCZ, compared to healthy subjects. Interestingly, our data revealed that the levels of the enzyme responsible for d-Ser biosynthesis, SRR,^7^ do not significantly change between control and SCZ-affected individuals in both the brain regions analyzed. This result is in line with unaltered SRR immunoreactivity found in another cohort of SCZ DLPFC samples,^27^ although other studies indicate either increased or decreased SRR protein levels in this brain region or in the hippocampus of patients.^4, 27, 28^ Considering the acknowledged relevance of *SRR* as a risk gene to SCZ vulnerability,^29^ future studies will be necessary to further investigate the SRR protein expression levels in the SCZ brain.

Differently from the co-agonist d-Ser, d-Asp, a d-amino acid highly enriched in the embryonic brain, acts as an endogenous agonist of NMDARs by stimulating the l-Glu binding sites.^9, 10, 30^ In addition, through the activation of presynaptic NMDARs, d-Asp also evokes the presynaptic release of cortical l-Glu in freely moving mice analyzed by in vivo microdialysis.^31^ Regarding d-Asp occurrence, we confirmed in a new *post-mortem* brain bank our earlier results evidencing an approximate 40% d-Asp levels reduction in the PFC of SCZ-affected patients.^18^ Indeed, herein we found a significant 30% decrease of this d-amino acid content in the DLPFC of SCZ patients compared to control cases. Such a reduction is selective for the DLPFC since we detected comparable levels of d-Asp in the hippocampus of SCZ-affected and healthy individuals. In both the brain regions analyzed, our study revealed that the content of d-Asp did not change over the age span of subjects, regardless of their clinical diagnosis.

Interestingly, in the DLPFC of SCZ-affected patients the reduced d-Asp content is linked to significantly increased (~25%) DDO enzymatic activity. This observation highlights, for the first time, the existence of a dysfunctional metabolic process at the level of DDO activity in the SCZ brain. Notably, our in vitro enzymatic assays support a putative direct pathophysiological dysfunction of d-Asp levels in SCZ since they exclude that the altered d-Asp catabolism in the DLPFC of patients could depend on secondary effect associated to previous antipsychotic medication. In fact, some of the most commonly used typical (chlorpromazine and haloperidol) or atypical (clozapine) antipsychotic drugs were unable to affect the activity of the recombinant hDDO. However, we cannot exclude that these drugs, rather than exerting a specific direct action on DDO, may influence d-Asp levels by cumulative effects on different neurotransmitter systems following a lifetime treatment. To our knowledge, this is the first report investigating the potential influence of antipsychotic drugs on DDO enzymatic activity; therefore, further investigations will be required to shed light on this important issue. Besides antipsychotic treatment, also the *post-mortem* delay (PMD)^32, 33^ and the age of donors represent potential confounding factors that could affect the tissue levels of amino acids. However, it is unlikely that the higher PMD observed in the brain samples of SCZ patients and their older age, compared to controls, may account for the reduced d-Asp levels found in the DLPFC since no significant differences in d-Asp concentration were detected in the hippocampus of the same subjects. Moreover, d-Asp levels did not correlate with age, PMD and pH, thus reducing the potential confounding effect of these variables. Nevertheless, a larger sample size would be needed to replicate our findings.

The altered DDO enzymatic activity detected in this cohort of *post-mortem* SCZ brains is reminiscent of the increased activity of the d-Ser-degrading enzyme, DAAO, previously found in the *post-mortem* brain of SCZ-affected subjects.^34, 35^ Altogether, these results suggest that an overall alteration of the enzymes controlling the catabolism of the two main neuroactive d-amino acids, i.e., DAAO and DDO, could contribute to NMDAR hypostimulation in SCZ. In agreement with an abnormal degradation of d-Ser in the SCZ brain, several findings demonstrated a genetic association between this neuropsychiatric illness and the gene *G72* (refs 2, 36), which encodes the protein pLG72 that interacts with DAAO and regulates its activity.^37^ In addition to genetic evidence, altered pLG72 protein levels have been found in the plasma of patients with SCZ, compared to control subjects.^38^ Unlike DAAO, the existence of putative modulators of DDO has never been investigated so far. Therefore, in the light of the dysfunctional DDO activity found in the DLPFC of SCZ patients, future studies will be mandatory to assess the occurrence of regulatory molecules and cellular factors that could tune the metabolic control played by DDO over d-Asp under physiological and pathologic conditions. In this regard, the recent pioneering research of novel inhibitors specific for hDDO^39^ well suits with the idea to reduce the DDO activity in SCZ brain in the attempt to increase the availability of d-Asp and potentiate, in turn, the overall NMDAR-mediated transmission.

Besides the altered DDO enzymatic activity observed in the DLPFC of patients with SCZ, our work did not reveal significant variations in the methylation state of *DDO* transcriptional regulatory region and in the *DDO* mRNA levels between SCZ-affected and control individuals. We also assessed the potential existence of copy number variations in the *DDO* gene but the genetic analysis did not evidence alterations in both diagnosis groups (see Supplementary Results and Supplementary Table 4). These data suggest that no gross molecular and genetic alterations occur in this cohort of SCZ cases until post-transcriptional regulatory level. However, the absence of reliable antibodies selective for DDO did not allow us to evaluate the absolute levels of this protein in the *post-mortem* human brains.

In addition to DDO, we also evaluated the potential contribution of SRR to the reduction of d-Asp levels observed in the DLPFC. Indeed, recent data evidenced that such d-Ser-producing enzyme could be also involved in d-Asp biosynthesis.^19, 20^ However, our results excluded that the d-Asp reduction found in the DLPFC could depend from altered SRR protein levels since our western blotting data revealed comparable amount of this protein between SCZ patients and healthy subjects.

In contrast to the decreased d-Asp levels found in the DLPFC of patients, no alteration in the hippocampus was observed, with the exception of an increase in *DDO* gene methylation, whose biological significance remains unclear. Remarkably, the selectivity of d-Asp reduction in the DLPFC and the reproducibility of this result in another *post-mortem* brain bank^18^ could overall suggest a greater vulnerability to d-Asp metabolic dysfunctions in a brain are acritically involved in the pathophysiology of SCZ. Indeed, several reports suggest the occurrence of functional and anatomical alterations in the DLPFC of SCZ-affected patients^40^ that are related to aberrant NMDAR-dependent signaling,^22^ and are likely responsible for the typical cognitive symptomatology of SCZ.^41^ In line with an involvement of the NMDAR agonist, d-Asp, in cortical processes relevant to SCZ, we previously found that a polymorphism in the human *DDO* gene (rs3757351), associated to reduced *DDO* mRNA levels (and, therefore, potentially yielding higher d-Asp levels), predicts greater in vivo prefrontal activity during working memory processing in healthy subjects.^17^ As for humans, research in preclinical models has been recently indicating a link between d-Asp content and cortical phenotypes related to SCZ. Indeed, we previously demonstrated that increased levels of this d-amino acid in knockout mice for the *Ddo* gene (*Ddo* ^-/-^) enhance dendritic length and spine density in cortical pyramidal neurons,^17^ potentiate cortico-hippocampal connectivity, and attenuate the functional activation of cortical areas induced by PCP.^16^ Moreover, oral d-Asp supplementation in mice improves cognitive and social interaction impairments produced by spared nerve injury of the sciatic nerve.^42^ Altogether, the beneficial synaptic and cognitive effects of d-Asp in preclinical models and its reduced concentration in the human SCZ *post-mortem* brain suggest that novel strategies, aimed at rising the cerebral availability of this endogenous NMDAR agonist, could be considered in future clinical studies in order to reverse the NMDAR dysfunctions observed in SCZ. The efficacy of d-Ser as add-on pharmacotherapy in the treatment of SCZ symptoms^3^ encourages to pursue similar clinical approaches also with d-Asp.

Our research also investigated the regulation of endogenous d-Asp levels in human DLPFC and hippocampus exerted by DDO, the only known enzyme involved in the catabolism of this d-amino acid.^9^ We confirmed in humans our previous evidence in the mouse brain about the pivotal control played by *DDO* gene methylation on *DDO* gene transcription.^8^ Indeed, our present data revealed that the regional changes in *DDO* gene methylation (higher in the DLPFC than in the hippocampus) are inversely related to the variations in *DDO* mRNA levels (lower in the DLPFC than in the hippocampus), regardless of the clinical diagnosis. However, we also found evidence that the regional diversity in *DDO* gene transcription does not help to explain the higher d-Asp levels found in the DLPFC, compared to the hippocampus. Indeed, the DDO enzymatic activity detected in the DLPFC and hippocampus is comparable in control subjects or even higher in the DLPFC of SCZ patients. The comprehension of the metabolic mechanisms controlling the endogenous synthesis of d-Asp in the brain will be necessary to understand more clearly the present observations and the potential pathophysiological significance of d-Asp dysregulation.

Differently from its low occurrence in adulthood, d-Asp reaches the highest levels in the brain during the embryonic phase.^1, 8^ Although the biological significance of d-Asp in prenatal phase is still unknown, it is tempting to hypothesize that such abundance could imply a functional involvement for this d-amino acid in glutamatergic neurotransmission since in this period NMDARs undergo a strict developmental regulation^43^ and critically affect fundamental cellular processes, including proliferation, migration, apoptosis, synaptogenesis, and differentiation.^44^ Moreover, it is noteworthy to mention that besides the mere direct effect produced by d-Asp on postsynaptic NMDARs, this d-amino acid can also trigger the release of cortical l-Glu through the stimulation of presynaptic NMDARs,^31^ thus probably acting as an “amplifier” of glutamatergic system. Since the current working model of SCZ postulates that this disorder has a neurodevelopmental origin linked to NMDAR hypofunction, we hypothesize that precocious alteration in d-Asp metabolism might significantly impact on NMDAR activity in developmental phase, when genetic abnormalities and aversive environmental factors are known to confer greater susceptibility to develop SCZ symptoms at adulthood.^45^ Unfortunately, the present study cannot establish whether the altered cortical d-Asp metabolism found in the DLPFC of SCZ patients has a neurodevelopmental onset or develops at later stages of life. In the light of our hypothesis, such dysfunction would acquire greater relevance if it already existed in the developing brain. The future generation of transgenic animal models with reduced prenatal levels of d-Asp will be a strategic tool for clarifying the neurobiological significance of this d-amino acid in the immature brain and, in turn, for better defining its potential implication in early NMDAR-dependent processes relevant to SCZ.

## Materials and methods

### Human tissue collection

DLPFC and hippocampus samples from *post-mortem* brains of non-psychiatrically ill individuals (Ctrl) and SCZ patients (*n* = 20/diagnosis/brain region) were obtained from The Human Brain and Spinal Fluid Resource Center (Los Angeles Healthcare Center, Los Angeles, CA, USA). All tissue collection and processing was carried out under the regulations and licenses of the Human Tissue Authority and in accordance with the Human Tissue Act of 2004. Clinical diagnosis of SCZ was performed according to DSMIII-R criteria. Demographic characteristics of control and SCZ subjects are described in Supplementary Table 1. Frozen tissues were pulverized in liquid nitrogen and stored at −80 °C for subsequent processings. All the following analyses were executed by experimenters blind to the clinical conditions of the subjects.

### High-performance liquid chromatography analysis

Brain tissue samples were analyzed as previously reported^46^ with minor modifications.^8^ Samples were homogenized in 1:20 (w/v) 0.2 M TCA, sonicated (three cycles, 10 s each) and centrifuged at 13,000 *g* for 20 min The precipitated protein pellets were stored at −80 °C for protein quantification, while the supernatants were neutralized with NaOH and subjected to pre-column derivatization with o-phthaldialdehyde/*N*-acetyl-l-cysteine. Diastereoisomer derivatives were resolved on a Simmetry C8 5-μm reversed-phase column (Waters, 4.6 × 250 mm). Identification and quantification of d-Ser, l-Ser, d-Asp, and l-Asp were based on retention times and peak areas, compared with those associated with external standards. The identity of d-Asp, l-Asp, and d-Ser peaks was confirmed by adding known amount of external standards, and by the selective degradation catalyzed by the RgDAAO M213R variant,^47^ StLASPO,^48^ and wild-type RgDAAO,^49^ respectively. The samples were added with 10 μg of the enzymes, incubated at 30 °C for 60 min and then derivatized. The amino acids total amount detected in homogenates was normalized by gram of tissue.

### DNA methylation analysis

Methylation status of the putative *DDO* promoter was assessed through a strategy based on the locus-specific amplification of bisulfite-treated genomic DNA, as described in Supplementary Methods.

### RNA extraction and quantitative RT-PCR analysis

Total RNA extraction and qRT-PCR were performed as described in Supplementary Methods. *DDO* mRNA expression levels were normalized to the mean of two housekeeping genes: *β-actin* and *cyclophilin* (*PPIA*). The following primers were used for cDNA amplification: *DDO-*fw 5′-GGTGTTCATTTGGTATCAGGTTG-3′ and *DDO-*rev 5′-CTTTCGAAATCCCAGAACCA-3′; *β-actin*-fw 5′-TCCTCCCTGGAGAAGAGCTA-3′ and *β-actin*-rev 5′-CGTGGATGCCACAGGACT-3′; *PPIA-*fw 5′-TTCATCTGCACTGCCAAGAC-3′ and *PPIA*-rev 5′-CACTTTGCCAAACACCACAT-3′. *DDO* mRNA expression was calculated using the relative quantification method (2^−ΔΔCt^).

### Enzymatic activity assay

DDO activity measurements in human *post-mortem* brains were performed by using the Amplex UltraRed fluorescent reagent (Invitrogen, ThermoFisher Scientific, Waltham, MA 0245, USA) adapting the procedure previously reported.^37, 50^ Detailed procedure is described in Supplementary Methods. DDO activity was normalized by the total protein content and expressed as μU/mg protein.

### In vitro inhibition assays

The effect of antipsychotic drugs on hDDO activity was evaluated in vitro using a coupled enzyme assay and the Amplex UltraRed reagent (Life Technologies, Carlsbad, CA USA). Detailed procedure is described in Supplementary Methods.

### Western blotting

Samples preparation and immunoblotting were performed as previously described.^18^ Frozen, powdered samples from *post-mortem* brains were sonicated in 1% SDS and boiled for 10 min Proteins were separated by sodium dodecyl sulfate polyacrylamide gel electrophoresis and electroblotted onto polyvinylidene difluoride membranes (GE-Healthcare). Immunodetection was accomplished by using anti-serine racemase (1:500, Santa Cruz Biotechnology, Santa Cruz, CA, USA) and anti-α-tubulin (1:50,000, Sigma, St Louis, MO, USA) antibodies. Blots were then incubated in horseradish peroxidase-conjugated secondary antibodies. Immunoreactivity signals were detected by enhanced chemiluminescence (GE-Healthcare) and quantified by Quantity One software (Bio-Rad). Optical density values were normalized to α-tubulin for variations in loading and transfer.

### Statistical analysis

Normal distribution assumption for continuous variables was checked by means of Q-Q plot and Shapiro–Wilks and Kolmogorov–Smirnov tests. We observed a non normal (or suspected non normal) distribution for d-/l-Asp and d-/l-Ser, both as absolute levels and as ratios, therefore, a non-parametric approach was used for all statistical analyses. Correlation between continuous variables was assessed using Spearman coefficient. Two-samples comparisons of continuous variables were performed using the Mann–Whitney *U*-test. A *p*-value < 0.05 was considered as statistically significant. All analyses were performed using SAS Release 9.4 (SAS Institute, Cary, NC, USA) and GraphPad (GraphPad Prism Software, Inc., La Jolla, CA, USA).

## Electronic supplementary material

Supplementary Information
