# Supplementary material for: Decreased free d-aspartate levels are linked to enhanced d-aspartate oxidase activity in the dorsolateral prefrontal cortex of schizophrenia patients
Source: NPJ Schizophr. 2017 Apr 6;3:16. doi: 10.1038/s41537-017-0015-7 (PMC5441530; doi:10.1038/s41537-017-0015-7)
Supplement: Supplementary file 1 — Supplementary Information [file 41537_2017_15_MOESM1_ESM.docx]

**Supplementary Information**

Decreased free D-aspartate levels are linked to enhanced D-aspartate oxidase activity in the dorsolateral prefrontal cortex of schizophrenia patients

Tommaso Nuzzo^1,2,^*, Silvia Sacchi^3,4,^*, Francesco Errico^1,5,^*, Simona Keller^5,6^, Orazio Palumbo^7^, Ermanno Florio^5,6^, Daniela Punzo^1,2^, Francesco Napolitano^1,5^, Massimiliano Copetti^8^, Massimo Carella^7^, Alessandro Bertolino^9^, Lorenzo Chiariotti^5,6^, Loredano Pollegioni^3,4,@^, Alessandro Usiello^1,2,@^

^1^ Laboratory of Behavioural Neuroscience, Ceinge Biotecnologie Avanzate, 80145, Naples, Italy; ^2^ Department of Environmental, Biological and Pharmaceutical Sciences and Technologies, Second University of Naples (SUN), 81100, Caserta, Italy; ^3^ Department of Biotechnology and Life Sciences, University of Insubria, 21100, Varese, Italy; ^4^ The Protein Factory Research Center, Politecnico di Milano and University of Insubria, 20133, Milan, Italy; ^5^ Department of Molecular Medicine and Medical Biotechnology, University of Naples “Federico II”, 80131 Naples, Italy; ^6^ Endocrinology and Molecular Oncology Institute (I.E.O.S.), National Research Council (C.N.R.); ^7^ Medical Genetics Unit, IRCCS Casa Sollievo della Sofferenza, 71013, San Giovanni Rotondo, FG, Italy; ^8^ Unit of Biostatistics, IRCCS Casa Sollievo della Sofferenza,71013, San Giovanni Rotondo, FG, Italy; ^9^ Department of Basic Medical Science, Neuroscience, and Sense Organs, University of Bari “Aldo Moro”, 70124, Bari, Italy.

**Supplementary Methods**

*Copy number analysis in human post-mortem samples*

The CytoScan HD assay was performed according to the manufacturer’s protocol, starting with 250 ng DNA extracted from dorsolateral prefrontal cortex (DLPFC) samples. Briefly, total genomic DNA was digested with a restriction enzyme (NspI), ligated to an appropriate adapter for the enzyme, and subjected to PCR amplification using a single primer. After digestion with DNase I, the PCR products were labeled with a biotinylated nucleotide analogue, using terminal deoxynucleotidyl transferase, and hybridized to the microarray.

The array contains more than 2,600,000 copy number variation (CNV) markers across the genome, including 750,000 genotype-able single nucleotide polymorphism (SNP) markers. The median inter-marker distance is less than 1 kb. Samples preparation, hybridization and scanning have been performed as previously described^1^. Hybridization was carried out in the Hybridization Oven 645 device while subsequent washing and staining were performed using the Fluidics Station 450. The array was then scanned with the Scanner 3000 7G and both quality control step and copy number analysis were performed using the Chromosome Analysis Suite Software version 3.1 (Affymetrix, Santa Clara, CA): (1) the raw data file (.CEL) was normalized using the default options; (2) an unpaired analysis was performed using as baseline 270 HapMap samples in order to obtain Copy numbers value from .CEL files while the amplified and/or deleted regions were detected using a standard Hidden Markov Model (HMM) method. A copy number variation was validated if an abnormal log2 ratio was obtained for at least 5 contiguous probes.

*DNA extraction and methylation analysis in humans*

Genomic DNA was prepared using DNeasy® Blood & Tissue Kit (Qiagen, Hilden, Germany), following the manufacturer’s instructions. DNA was quality checked by 260 nm/280 nm absorbance ratio using NanoDrop 2000 (Thermo Scientific) and it was quantified using Qubit® 2.0 Fluorometer with the dsDNA broad range assay kit (Invitrogen, Q32850). Bisulfite treatment was performed using EZ DNA Methylation Kit (Zymo Research, USA). To evaluate the DNA methylation levels, a double step PCR strategy was used to generate an amplicon library of bisulfite treated DNA that was sequenced by Illumina Miseq Sequencer as previously described^2^. Sequence of the bisulfite-specific primers used for the analysis were: *Ddo* Fw 5’- aTTtaTaaatTagTtggagaaagTTTag -3’ (nt position from -227 to -200)*; Ddo* Rv 5’- cctattcaAacacactcccaaactcc- 3’ (nt position from +145 to +171)*.* Sequences in FASTQ format by Illumina sequencing machine were processed as previously described^2^.

*RNA extraction and quantitative RT-PCR analysis*

Total RNA was extracted from *post-mortem* tissues using miRNeasy® kit (Quiagen, Hilden, Germany) according to the manufacturer’s instructions. RIN of samples was assessed using Agilent 2100 Bioanalyzer Expert (Santa Clara, California, USA) and Biorad Experion Automated electrophoresis Station (Hercules, CA) prior to cDNA synthesis with Transcriptor First Strand cDNA Synthesis kit (Roche Diagnostics, Mannheim, Germany). Total RNA (0.5 μg per sample) was used to synthesize cDNA. Quantitative RT-PCR with Real Time ready catalog Assays (Roche Diagnostics) and LightCycler® 480 Probe Master (Roche Diagnostics) was performed on a Light Cycler 480 Real Time PCR system with 96-well format (Roche Diagnostics). All measurements from each subject were performed in duplicate.

*Enzymatic activity assay*

DDO activity measurements in human *post-mortem* brains were performed by using the Amplex UltraRed fluorescent reagent (Invitrogen, Waltham, MA, USA) adapting the previously reported procedure^3,4^. DLPFC and hippocampus samples were homogenized in lysis buffer (50 mM sodium phosphate, pH 8.0, 5 µM FAD, 0.1% v/v Triton X-100, 0.7 µg/mL pepstatin, 1 µg/mL leupeptin), incubated 10 min on ice, sonicated (5 cycles, 20 s + 30 s on ice each) and centrifuged at 13,000 g for 30 min at 4 °C. 150 µL of the different supernatants (tissue extracts) were added with 50 µL of the activity assay solution containing 74.4 µM Amplex UltraRed, 0.2 units/ml horseradish peroxidase, 10 mM NaN_3_, 5 µM FAD, 25 mM D-Asp and incubated for 6 h at room temperature in the dark. Controls without tissue extract samples were assayed simultaneously; H_2_O_2_ production, indicating DDO activity, was measured as the difference in fluorescence emission between samples and controls assay mixtures. A calibration curve was obtained by adding known amounts of recombinant human DDO (hDDO) to lysis buffer (0.025–100 µU range). Furthermore, 10 mM potassium sodium tartrate (a DDO inhibitor) was added to verify whether the observed fluorescence changes were effectively due to DDO activity. Following the determination of the total protein concentration of tissue extracts by the Bradford assay method, DDO activity was normalized by the total protein content of the different samples and expressed as μU/mg protein.

*In vitro inhibition assays*

Recombinant D-aspartate oxidase from hDDO origin was overexpressed in *E. coli* cells and purified as previously reported^5^, with minor modifications. The effect of antipsychotic drugs on hDDO activity was evaluated *in vitro* using a coupled enzyme assay and the Amplex UltraRed reagent (Life Technologies, Carlsbad, CA USA) ^6,7^. Stock solutions were prepared by dissolving drugs in DMSO, and subsequently diluted in 50 mM sodium phosphate, pH 7.4. Vehicle concentration was carefully maintained such that all experimental groups had the same DMSO concentration (never exceeding 1.5%). All enzymatic assays were conducted at room temperature in 96-well plate format using an automated liquid-handler system (epMotion 5075; Eppendorf, Hamburg, Germany). Briefly, 0.03 U/mL of hDDO were incubated for 30 min with the different compounds to be tested (0-1250 μM concentration range) in the presence of exogenous FAD (4 µM). Then, 7.5 mM D-Asp, 0.1 U/mL horseradish peroxidase (Roche, Basel, Switzerland) and 35 μM Amplex UltraRed reagent were added and the fluorescence of oxidized reagent produced by hDDO activity was recorded after 30 min (540 and 595 nm: excitation and emission wavelengths, respectively). Data were expressed as relative activity (%): the emission value in the absence of drugs was set as 100% activity.

*Animals*

Three/four-month-old C57BL/6J male mice were purchased from Jackson Laboratory (Bar Harbour, ME, USA). Animals were group housed (five per cage), at a constant temperature (22±1 °C) on a 12 h light/dark cycle (lights on at 7 AM) with food and water *ad libitum*. All research involving animals was carried out in accordance with the European directive 86/609/EEC governing animal welfare and protection, and was approved by the Animal Welfare and Ethical Review Body Committee (AWERBC) of Ceinge Biotecnologie Avanzate.

*Mouse tissue collection*

Mice were killed and the prefrontal cortex and hippocampus were dissected out within 20 s on an ice-cold surface. All tissue samples were pulverized in liquid nitrogen and stored at -80 °C for subsequent processing.

*Methylation analysis in mice*

The analysis of mouse *Ddo* gene methylation was performed as previously described^2^. DNA from frozen brain tissue samples was extracted using the ZR Genomic DNA tissue Midi Prep kit (Zymo Research). The integrity and the amount of genomic DNA were assessed by 0.8% agarose gel electrophoresis and Qubit Fluorometric Quantitation (Invitrogen), respectively. Sodium bisulfite conversion was performed using EZ DNA Methylation Kit (Zymo Research) following the manufacturer’s instruction. Methylation status was assessed by Illumina MiSeq sequencing. The sequence of the bisulfite-specific primers used for this analysis were: *Ddo* Mouse Fw 5’-gtgtgtttTtgaggaggtgaTaTtTa-3’ (nt position from -468 to -444) and *Ddo* Mouse Rv 5’-aActtaccctccattAAtccatAcc-3’ (nt -88 to -63). The capital letters in the primers sequences indicate the original C or G, respectively. Sequences in FASTQ format by Illumina sequencing machine were processed as described in Punzo et al. (2015)^2^. Data were analyzed by Student’s *t* test.

*Quantitative RT-PCR in mice*

Detection of mouse *Ddo* mRNA levels was performed according to a previous protocol^2^. Total RNA was isolated using chloroform-isopropanol (TRIZOL RNA Isolation Protocol) method according to the manufacturer's instructions. The integrity of the RNA was assessed by denaturing agarose gel electrophoresis (presence of sharp 28S, 18S and 5S bands) and spectrophotometry. Total RNA was purified to eliminate potentially contaminating genomic DNA using recombinant DNAse. One μg of total RNA of each sample was reverse-transcribed with QuantiTect(R) Reverse Transcription (Qiagen) using an optimized blend of oligo-dT and random primers according to the manufacturer's instructions. qPCR amplifications were performed using LightCycler® 480 SYBR Green I Master (Roche Diagnostic GmbH, Mannheim, Germany) in a LightCycler® 480 Real Time thermocycler (Roche). The following protocol was used: 10 s for initial denaturation at 95.0 °C followed by 40 cycles consisting of 10 s at 94.0 °C for denaturation, 10 s at 60.0 °C for annealing temperature and 6 s for elongation at 72.0 °C. The following primers were used for *Ddo* cDNA amplification: *Ddo* fw 5’-CCTGGTATCTGGTTGGCAGA-3’ and *Ddo* rev 5’- TCGAAATCCCAGCACCACAT-3’; *β-actin* gene was used as housekeeping gene for PCR reaction: *β-actin* fw 5’- CTAAGGCCAACCGTGAAAAGAT-3’ and *β-actin* rev 5’- GCCTGGATGGCTACGTACATG-3’. Data were analyzed by Student’s *t* test.

**Supplementary Results**

**Copy number analysis of the genes involved in D-aspartate and D-serine metabolism**

As a first point, we used the *post-mortem* tissues of patients with SCZ and control subjects to analyze the potential existence of CNVs in the *DDO* gene (chromosome 6q21), and in the genes encoding for the enzymes and regulatory protein responsible for the metabolism of D-Ser, such as *D-amino acid oxidase* (*DAAO*, chromosome 12q24.11), *G72* (chromosome 13q33.2) and *serine racemase* (*SR*, chromosome 17p13.3)^8,9^. To this aim, we performed a high-resolution chromosomal microarray analysis on the DNA extracted by the *post-mortem* DLPFC samples by using the CytoScan HD array platform. As results, no CNVs encompassing the *DDO* gene, as well as *DAAO*, *G72* and *SR* genes, have been identified in both class of samples enrolled for the study (Supplementary Table 4).

**Regional analysis of *Ddo* gene methylation and *Ddo* gene transcription in the mouse brain**

As first point, we assessed whether the methylation degree in the putative promoter region of the *Ddo* gene^2^ changes depending from the brain region analyzed. In line with the methylation study performed in humans (see main text), we focused our analysis in the mouse prefrontal cortex (PFC) and hippocampus. As in a previous work^2^, DNA methylation analysis was assessed through a strategy based on the locus-specific amplification of bisulfite-treated genomic DNA.

We covered the genomic region upstream the transcription start site (TSS) spanning nucleotides -443 to +88 and including 6 CpG sites (positions -363, -330, -318, -242, -175, -125) (Supplementary Figure 1a), and analyzed their average methylation state. Interestingly, in line with the results obtained in human *post-mortem* tissues, we found significantly higher methylation in the PFC, compared to the hippocampus (mean values: PFC, 25.5%; hippocampus, 18.0%; *p* < 0.0001, Student’s *t* test) (Supplementary Figure 1b).

Then we evaluated the transcription of *Ddo* gene in these two brain regions of mice through qRT-PCR. In line with the differential methylation profile observed above, the statistical analysis evidenced that the *Ddo* mRNA levels are lower in the mouse PFC than in the hippocampus (mean values: PFC, 0.90 ± 0.04; hippocampus, 1.13 ± 0.04; *p* < 0.01, Student’s *t* test) (Supplementary Figure 1c).

**Supplementary Table 1.** Demographic and clinical characteristics of deceased non-psychiatric (control) subjects and schizophrenia-affected patients. Tissue samples were obtained from The Human Brain and Spinal Fluid Resource Center (VA West Los Angeles Healthcare Center, Los Angeles, CA 90073, USA). Gender, age, *post-mortem* delay, pH and clinical diagnosis of each subject are indicated. The antipsychotic drugs used by schizophrenia-affected patients are also reported. The total number of subjects, the number of males and females analyzed in each diagnosis group, as well as the age, PMD and pH means (± SEM) are reported at the end of the list. Statistical analyses indicated non significant difference between patients with schizophrenia and controls for gender (*p*=0.1675, χ^2^ test) and pH (*p*=0.3658, Mann-Whitney test), while the age and the PMD are significantly higher in schizophrenia-affected subjects compared to healthy individuals, (*p*=0.0004 and *p*=0.0465, respectively, Mann-Whitney test). Nr = number, PMD = *post-mortem* delay, M = male, F = female, CA = carcinoma, COPD = chronic obstructive pulmonary disease, CVA = cerebrovascular accident.

|  | **Control** | | | | | | | |  |
| --- | --- | --- | --- | --- | --- | --- | --- | --- | --- |
|  | Nr | Gender | Age (years) | PMD (h) | pH | Clinical Diagnosis | Antipsychotic medication | |  |
|  | 1 | M | 47 | 12.5 | 6.53 | CA (esophagus) with metastases to the liver | No | |  |
|  | 2 | M | 66 | 17.3 | 6.49 | CA (lung), COPD | No | |  |
|  | 3 | F | 92 | 23.3 | 6.75 | CA (uterus, stomach), Congestive hearth failure, Hypertension, Macular degeneration, | No | |  |
|  | 4 | M | 84 | 11.8 | 6.79 | CA (stomach), Renal failure, acute, COPD | No | |  |
|  | 5 | M | 70 | 11.8 | 6.62 | Coronary hearth disease, Leukemia, Type I diabetes, Myocardial infarction, Congestive | No | |  |
|  | 6 | M | 87 | 9.3 | 6.76 | Congestive hearth failure, Atherosclerosis, COPD | No | |  |
|  | 7 | M | 58 | 9.0 | 6.32 | CA (colon) | No | |  |
|  | 8 | M | 68 | 10.5 | ND | CA (lung), Alcohol abuse, Type I diabetes, Transient Ischemic Attack | No | |  |
|  | 9 | M | 80 | 14.0 | 6.49 | CA (bladder), Hypertension, Diabetes type II, CVA | No | |  |
|  | 10 | M | 76 | 16.0 | 6.55 | CA (lung). Pulmonary emphysema | No | |  |
|  | 11 | M | 75 | 11.5 | 6.6 | CA (prostate) Coronary hearth disease, Hypertension | No | |  |
|  | 12 | M | 66 | 13.3 | ND | CA (larynx) Metastasis to bone and liver, Type I diabetes | No | |  |
|  | 13 | M | 64 | 17.5 | 6.63 | Lymphoma, Coronary Artery Disease | No | |  |
|  | 14 | M | 80 | 12.0 | ND | CA (kidney) Hypertension, Atrial fibrillation, Macular degeneration, COPD | No | |  |
|  | 15 | F | 83 | 17.6 | 6.41 | CA (breast, uterus, colon), Macular degeneration, Chronic urinary tract infection | No | |  |
|  | 16 | F | 79 | 14.0 | ND | Coronary hearth disease, Hypertension | No | |  |
|  | 17 | M | 61 | 19.5 | 6.29 | Normal | No | |  |
|  | 18 | M | 70 | 12.0 | ND | Renal failure, acute, Diabetes type I | No | |  |
|  | 19 | M | 72 | 12.2 | 6,54 | COPD, Pulmonary emphysema | No | |  |
|  | 20 | F | 81 | 14.5 | ND | COPD, Pneumonia, Osteoporosis, Tuberculosis | No | |  |
|  | **Tot.=20** | **16M/4F** | **72.9±2.4** | **14.0±0.8** | **6.56±0.15** |  |  | |  |
| **Schizophrenia** | | | | | | | | |  |
|  | Nr | Gender | Age (years) | PMD (h) | pH | Clinical Diagnosis | | Antipsychotic medication |  |
|  | 21 | M | 46 | 21.7 | 6.45 | Schizophrenia, Depression, Bipolar, Seizure disorder, Epilepsy, Substance abuse | | Quietapine |  |
|  | 22 | M | 55 | 10.7 | ND | Schizophrenia, Suicide, Overdose, Depression, Anxiety, Hypochondriasis | | Risperidone, Fluphenazine |  |
|  | 23 | M | 53 | 20.5 | ND | Schizophrenia, Depression, Bipolar, CA (lung) Paranoia, Psychosis, Hypertension, Anxiety | | Olanzapine, Fluphenazine |  |
|  | 24 | M | 70 | 24.0 | 6.56 | Schizophrenia, Paranoia, Aggressive behavior, Dementia, Impulse disorder, Tuberculosis | | ND |  |
|  | 25 | M | 35 | 35.7 | 6.51 | Schizophrenia, Alcohol abuse | | Risperidone, Haloperidol |  |
|  | 26 | F | 32 | 12.3 | 6.51 | Schizophrenia, Alcohol abuse history | | ND |  |
|  | 27 | M | 61 | 28.0 | 6.73 | Schizophrenia, Aggressive behavior, Suicide, Attempts, Anxiety, Tobacco abuse, Asthma | | Thioridazine, Fluphenazine, Quietapine, Paliperidone |  |
|  | 28 | F | 41 | 20.8 | 6.39 | Schizophrenia, Suicide, Stabbing, Psychosis, Disassociated Disorders, Electroconvulsive | | Quietapine, Haloperidol, Risperidone |  |
|  | 29 | F | 50 | 13.7 | ND | Schizophrenia, Suicide, Overdose, Aggressive behavior, Depression, Migraine, Hallucination | | Risperidone |  |
|  | 30 | M | 18 | 26.3 | 6.72 | Schizophrenia, Mentally retarded (clinical only), Attention Deficit Disorder, Sleep Apnea | | Risperidone |  |
|  | 31 | M | 46 | 11.6 | 6.41 | Schizophrenia, Suicide, hanging, Alcohol abuse, Substance abuse (not Alcohol), Depression | | Quietapine, Risperidone |  |
|  | 32 | F | 29 | 27.3 | 6.4 | Schizophrenia, Depression, Seizure Disorder, Attention Deficit Disorder, Aggressive behavior | | Aripiprazole, Risperidone |  |
|  | 33 | M | 60 | 10.3 | ND | Schizophrenia, Depression, Bipolar, Alcohol abuse, Psychotic disorder, Inappropriate sexual | | Ziprasidone, Risperidone |  |
|  | 34 | F | 75 | 14.9 | ND | Schizophrenia, CA (pancreas), Dementia, Therapeutic lobotomy, Dysphagia | | Risperidone |  |
|  | 35 | F | 77 | 14.7 | 6.38 | Schizophrenia, Alcohol abuse, Depression, Bipolar and Seizure disorder | | Fluphenazine, Risperidone |  |
|  | 36 | M | 77 | 26.5 | 6,56 | Schizophrenia, Hypertension, Dementia, COPD | | Quietapine |  |
|  | 37 | M | 24 | 12.8 | 6.47 | Depression, Schizophrenia, Substance abuse (not Alcohol), Suicide Attempts, Alcohol abuse | | Aripiprazole, Quietapine |  |
|  | 38 | F | 62 | 12.2 | 6.67 | Schizophrenia, Stroke/CVA, Depression, Hypertension, Diabetes type I | | Quietapine |  |
|  | 39 | F | 52 | 15.6 | 6.49 | Schizophrenia, Depression, CA (pancreas), Diabetes Type II, Hypothyroidism, Hypertension | | Compazine, Risperidone |  |
|  | 40 | M | 55 | 12.6 | ND | Schizophrenia, Paranoid Schizophrenia, Infection Bacterial, Psychotic disorder, Hallucination | | Fluphenazine, Chlorpromazine |  |
|  | **Tot.=20** | **12M/8F** | **50.9±3.9** | **18.6±1.6** | **6.52±0.12** |  | |  |  |

**­ Supplementary Table 2.** Mean values of D-aspartate, D-serine, L-aspartate, L-serine (expressed as nmol/g tissue), D-/total aspartate and D-/total serine ratios (expressed as %) are compared between control subjects (Ctrl) and schizophrenia-affected patients (SCZ) in the dorsolateral prefrontal cortex and hippocampus. *p* values are indicated for each comparison (Mann-Whitney test). Statistically significant *p* values are underlined.

|  | **Amino acid** |  | **Dorsolateral Prefrontal Cortex** | | | |  | **Hippocampus** | | | |  |
| --- | --- | --- | --- | --- | --- | --- | --- | --- | --- | --- | --- | --- |
|  |  |  | *Ctrl* | *vs* | *SCZ* | *p value* |  | *Ctrl* | *vs* | *SCZ* | *p value* |  |
|  | *D-aspartate (nmol/g tissue)* |  | 14.5 ± 1.1 |  | 10.2 ± 1.2 | 0.0117 |  | 7.8 ± 1.15 |  | 6.2 ± 0.1 | 0.4076 |  |
|  | *L-aspartate (nmol/g tissue)* |  | 1718.1 ± 112.3 |  | 1929.2 ± 170.9 | 0.2807 |  | 2134.3 ± 153.6 |  | 2466.6 ± 152.6 | 0.1150 |  |
|  | *D-/Total aspartate (%)* |  | 0.8 ± 0.1 |  | 0.6 ± 0.1 | 0.0002 |  | 0.4 ± 0.1 |  | 0.3 ± 0.1 | 0.1515 |  |
|  | *D-serine (nmol/g tissue)* |  | 167.0 ± 9.1 |  | 198.0 ± 13.2 | 0.0325 |  | 193.9 ± 11.1 |  | 190.5 ± 18.4 | 0.6922 |  |
|  | *L-serine (nmol/g tissue)* |  | 977.0 ± 93.7 |  | 1095.5 ± 84.3 | 0.2988 |  | 2051.6 ± 139.5 |  | 2467.0 ± 175.8 | 0.0905 |  |
|  | *D-/Total serine (%)* |  | 16.1 ± 1.2 |  | 15.9 ± 0.8 | 0.8356 |  | 9.4 ± 0.9 |  | 7.1 ± 0.4 | 0.0713 |  |
|  | *L-aspartate/L-serine* |  | 1.76 |  | 1.76 |  |  | 1.04 |  | 1.00 |  |  |

**Supplementary Table 3.** Mean values of D-aspartate, D-serine, L-aspartate, L-serine (expressed as nmol/g tissue), D-/total aspartate and D-/total serine ratios (expressed as %) are compared between the dorsolateral prefrontal cortex (DLPFC) and hippocampus in control subjects and schizophrenia-affected patients. *p* values are indicated for each comparison (Mann-Whitney test). Statistically significant *p* values are underlined.

|  | **Amino acid** |  | **Control** | | | |  | **Schizophrenia** | | | |  |
| --- | --- | --- | --- | --- | --- | --- | --- | --- | --- | --- | --- | --- |
|  |  |  | *DLPFC* | *vs* | *Hippocampus* | *p value* |  | *DLPFC* | *vs* | *Hippocampus* | *p value* |  |
|  | *D-aspartate (nmol/g tissue)* |  | 14.5 ± 1.1 |  | 7.8 ± 1.1 | 0.0003 |  | 10.2 ± 1.2 |  | 6.2 ± 0.1 | 0.0205 |  |
|  | *L-aspartate (nmol/g tissue)* |  | 1718.1 ± 112.3 |  | 2134.3 ± 153.6 | 0.0543 |  | 1929.2 ± 170.9 |  | 2466.6 ± 152.6 | 0.0250 |  |
|  | *D-/Total aspartate (%)* |  | 0.8 ± 0.1 |  | 0.4 ± 0.1 | < 0.0001 |  | 0.6 ± 0.1 |  | 0.3 ± 0.1 | 0.0153 |  |
|  | *D-serine (nmol/g tissue)* |  | 167.0 ± 9.1 |  | 193.9 ± 11.1 | 0.0758 |  | 198.0 ± 13.2 |  | 190.5 ± 18.4 | 0.5965 |  |
|  | *L-serine (nmol/g tissue)* |  | 977.0 ± 93.7 |  | 2051.6 ± 139.5 | < 0.0001 |  | 1095.5 ± 84.3 |  | 2467.0 ± 175.8 | < 0.0001 |  |
|  | *D-/Total serine (%)* |  | 16.1 ± 1.2 |  | 9.4 ± 0.9 | 0.0001 |  | 15.9 ± 0.8 |  | 7.1 ± 0.4 | < 0.0001 |  |

**Supplementary Table 4.** Copy number state for the *DDO* gene (1 = deletion; 2 = normal; 3 = duplication) obtained by the SNP-Array analysis performed on control (n = 19) and SCZ-affected subjects (n = 20) using the Cytoscan HD Array (Affymetrix, Santa Clara, CA). Base pair (bp) position were derived from the University of California Santa Cruz (UCSC) Genome Browser (http://genome.ucsc.edu/cgi-bin/hgGateway), build GRCh37 (hg19).

|  | **Gene** | **Chromosome** |  | **From probe (position, bp/hg19)** |  | **To probe (position, bp/hg19)** |  | **Copy Number State**  **Ctrl SCZ** | |  |
| --- | --- | --- | --- | --- | --- | --- | --- | --- | --- | --- |
|  | *D-aspartate oxidase* (*DDO*) | 6 |  | C-7MCLO (110,713,633) |  | C-7QQGZ (110,738,579) |  | 2 | 2 |  |
|  | *Serine racemase* (*SR*) | 17 |  | C-6PKHR (2,212,553) |  | C-7ECMQ (2,230,249) |  | 2 | 2 |  |
|  | *D-amino acid oxidase* (*DAAO*) | 12 |  | S-3MWDZ (109,271,928) |  | C-5MVLY (109,290,378) |  | 2 | 2 |  |
|  | *D-amino acid oxidase activator* (*PLG72/G72/DAOA*) | 13 |  | C-7HRXL (106,115,602) |  | C-5ZNVG (106,145,547) |  | 2 | 2 |  |

**
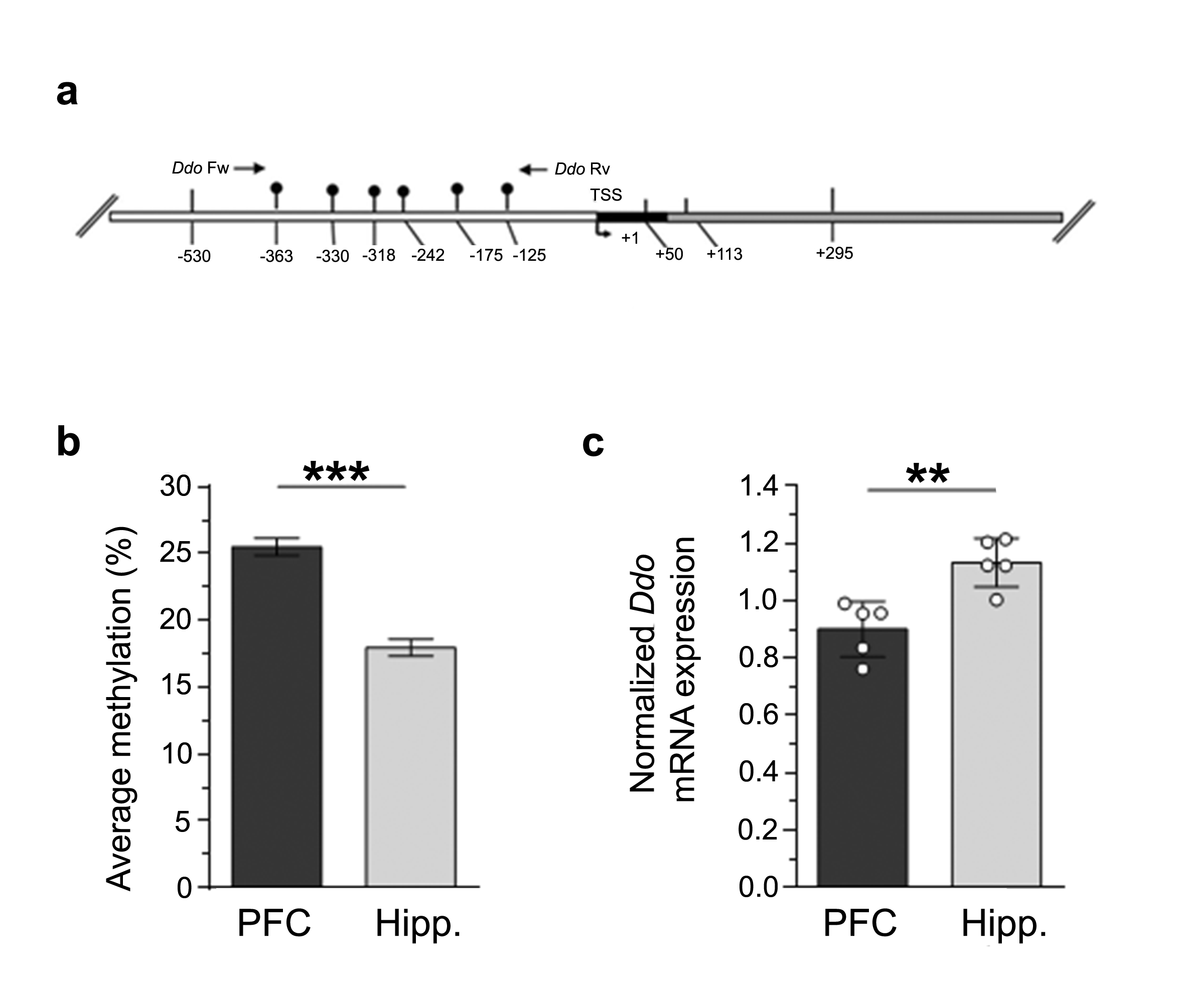
**

**Supplementary Figure 1.** *Ddo* gene methylation and gene transcription in the prefrontal cortex and hippocampus of mouse brain. **(a)** Structure of the putative mouse *Ddo* gene promoter. Arrow indicates the transcription start site (TSS, +1). White box represents the putative regulatory upstream region. Black box represents exon 1. Gray box represents first intron. Position of CpG sites is indicated as relative to TSS. Position of the primers used for bisulfite analysis is indicated by arrows at the top of the map (*Ddo* Fw, *Ddo* Rv). **(b)** Average methylation degree of the six CpG sites included in the regulatory region of *Ddo* in the prefrontal cortex (PFC) and hippocampus (Hipp.) of C57BL6/J mice. The percent (%) of methylation represents the average of the 6 CpG sites contained in the analyzed region. **(c)** Relative *Ddo* mRNA expression, measured by quantitative RT-PCR in the same brain regions of C57BL/6J mice. The *DDO* mRNA expression is normalized to *β-actin* gene and expressed as arbitrary units. Brain samples used for the average methylation analysis are the same as that used to measure *Ddo* mRNA relative expression (n = 5/brain region). Data are mean ± SEM. ** *p* < 0.01 (Student’s *t* test).

**Supplementary References**

1 Palumbo, O. *et al.* TBR1 is the candidate gene for intellectual disability in patients with a 2q24.2 interstitial deletion. *Am J Med Genet A* **164A**, 828-833, doi:10.1002/ajmg.a.36363 (2014).

2 Punzo, D. *et al.* Age-Related Changes in D-Aspartate Oxidase Promoter Methylation Control Extracellular D-Aspartate Levels and Prevent Precocious Cell Death during Brain Aging. *J Neurosci* **36**, 3064-3078, doi:10.1523/JNEUROSCI.3881-15.2016 (2016).

3 Sacchi, S. *et al.* pLG72 modulates intracellular D-serine levels through its interaction with D-amino acid oxidase: effect on schizophrenia susceptibility. *J Biol Chem* **283**, 22244-22256, doi:10.1074/jbc.M709153200 (2008).

4 Cappelletti, P., Campomenosi, P., Pollegioni, L. & Sacchi, S. The degradation (by distinct pathways) of human D-amino acid oxidase and its interacting partner pLG72--two key proteins in D-serine catabolism in the brain. *FEBS J* **281**, 708-723, doi:10.1111/febs.12616 (2014).

5 Katane, M. *et al.* Comparative characterization of three D-aspartate oxidases and one D-amino acid oxidase from Caenorhabditis elegans. *Chem Biodivers* **7**, 1424-1434, doi:10.1002/cbdv.200900294 (2010).

6 Hopkins, S. C. *et al.* Structural, kinetic, and pharmacodynamic mechanisms of D-amino acid oxidase inhibition by small molecules. *J Med Chem* **56**, 3710-3724, doi:10.1021/jm4002583 (2013).

7 Terry-Lorenzo, R. T. *et al.* Novel human D-amino acid oxidase inhibitors stabilize an active-site lid-open conformation. *Biosci Rep* **34**, doi:10.1042/BSR20140071 (2014).

8 Pollegioni, L. & Sacchi, S. Metabolism of the neuromodulator D-serine. *Cell Mol Life Sci* **67**, 2387-2404, doi:10.1007/s00018-010-0307-9 (2010).

9 Sacchi, S. D-Serine metabolism: new insights into the modulation of D-amino acid oxidase activity. *Biochem Soc Trans* **41**, 1551-1556, doi:10.1042/BST20130184 (2013).
